# Brain Atrophy as an Outcome of Disease-Modifying Therapy for Remitting-Relapsing Multiple Sclerosis

**DOI:** 10.1155/2023/4130557

**Published:** 2023-08-31

**Authors:** Magdalena Chylińska, Jakub Komendziński, Adam Wyszomirski, Bartosz Karaszewski

**Affiliations:** Department of Adult Neurology, Gdańsk Medical University, Gdańsk, Poland

## Abstract

**Introduction:**

Currently, clinical trials of DMTs strive to determine their effect on neuroinflammation and neurodegeneration. We aimed to determine the impact of currently used DMTs on brain atrophy and disability in RRMS. The main goal of this review is to evaluate the neuroprotective potential of MS therapy and assess its impact on disability.

**Methods:**

We performed a systematic analysis of clinical trials that used brain atrophy as an outcome or performed post hoc analysis of volumetric MRI parameters to assess the neuroprotective potential of applied therapies. Trials between 2008 and 2019 that included published results of brain parenchymal fraction (BPF) change and brain volume loss (BVL) in the period from baseline to week 96 or longer were considered.

**Results:**

Twelve from 146 clinical trials met the inclusion criteria and were incorporated into the analysis. DMTs that presented a large reduction in BVL also exhibited robust effects on clinical disability worsening, e.g., alemtuzumab with a 42% risk reduction in 6-month confirmed disability accumulation (*p* = 0.0084), ocrelizumab with a 40% risk reduction in 6-month confirmed disability progression (*p* = 0.003), and other DMTs (cladribine and teriflunomide) with moderate influence on brain atrophy were also associated with a marked impact on disability worsening. Dimethyl fumarate (DEFINE) and fingolimod (FREEDOMS I) initially exhibited significant effect on BVL; however, this effect was not confirmed in further clinical trials: CONFIRM and FREEDOMS II, respectively. Peg-IFN-*β*1a shows a modest effect on BVL and disability worsening.

**Conclusion:**

Our results show that BVL in one of the components of clinical disability worsening, together with other variables (lesion volume and annualized relapse rate). Standardization of atrophy measurement technique as well as harmonization of disability worsening and progression criteria in further clinical trials are of utmost importance as they enable a reliable comparison of neuroprotective potential of DMTs.

## 1. Introduction

Multiple sclerosis (MS) is a multicomponent disease characterized by inflammation, neurodegeneration, and failure of central nervous system repair mechanisms [1]. Early treatment is critical and helps reduce deterioration of physical ability and cognitive decline. The efficacy of disease-modifying therapies (DMTs) can be measured clinically, using the Expanded Disability Status Scale (EDSS) and Multiple Sclerosis Functional Composite (MSFC). Disability worsening could be defined as an increment in the EDSS score confirmed at 12 or 24 weeks of therapy [2]. Routine radiological evaluation of DMTs' efficacy consists of a variety of magnetic resonance imaging (MRI) methods (for example, T2 lesion volume, number of new and enlarging T2 lesions, and number of gadolinium-enhanced T1 lesions) mainly to evaluate neuroinflammation. Pathogenetic processes, neuroinflammation, and neurodegeneration develop independently and overlap each other in the early stages of the disease. Inflammation caused by transendothelial migration of leucocytes into the central nervous system is observed in MRI as active (gadolinium-enhancing) lesions [3]. A neurodegenerative process in MS caused by axonal transection and neuronal loss, leading to progressive atrophy of the brain tissue, can be found not only in lesions but also in normal-appearing white and gray matter (NAWM and NAGM, respectively) [3].

In the early stages of the disease, acute axonal damage and transection are associated with inflammation, especially with macrophage infiltration [4] and CD8 lymphocytes-mediated axon injury [5]. In chronic demyelinated lesions with little or no evidence of inflammation, axonal injury and axonal loss are present, suggesting that myelin-derived trophic support is of great importance for axonal survival [4]. Extensive evidence indicates that ion overload, iron dysregulation, mitochondrial dysfunction, and glutamate excitotoxicity play important roles in axonal destruction [6]. Assuming that axons constitute 46% of white matter (WM) volume, atrophy of the brain/loss of brain volume is a relevant exponent of axonopathy in MS [7]. Both histologic and neuroimaging studies have demonstrated that axonopathy plays a prominent role in the development of permanent functional deterioration [7].

Assessment of the impact of MS therapy on both inflammation and neurodegeneration can lead to the optimization of treatment options. Currently, an objective of MS therapy is to achieve a state of no evidence of disease activity (NEDA) [8]. Precise monitoring of the effectiveness of the applied therapy comprises no new/enlarged T2-lesions or gadolinium-enhancing lesions, no relapses, and no disability progression (according to the EDSS score) and is considered under the NEDA-3 criteria. The NEDA-4 criteria involve the components of NEDA-3 and a fourth criterion of ≤0.4% annualized brain volume loss (BVL) [9]. The anti-inflammatory profile of a given DMT may be obtained from a number of clinical trials and is easily estimated by the number of new T2-lesions, newly enlarged T2-lesions, and gadolinium-enhancing lesions. In contrast, the neuroprotective properties of DMTs are more difficult to evaluate. Neurodegeneration in MS as revealed in MR-based studies of brain atrophy has been observed since the earliest stages of the disease [10]. Brain atrophy measurements are influenced by biological factors such as inflammation, age, life habits, genetic load, comorbidities, and technical factors (e.g., quality of image acquisition, scanner and software changes, and pulse sequence) [11]. Annual BVL is accelerated in patients with relapsing-remitting multiple sclerosis (RRMS), ranging from 0.5% to 1.35%. By comparison, BVL in healthy individuals is 0.1-0.3% per year [12]. Anti-inflammatory effects of DMTs may cause resolution of edema and a decrease in the number or volume of inflammatory cells (i.e., microglia) [13]. This eventually leads to brain volume reduction, which is known as “pseudo-atrophy” and may last up to a year from the onset of therapy [14], prompting the typical 6-24 month period for the measurement of brain atrophy in clinical trials. Widespread MRI methods allow global and regional brain volume measurement, follow-up of brain volume changes over time, and estimation of the neuroprotective effect of DMT [11].

Many cross-sectional and longitudinal MRI techniques have been developed to evaluate brain atrophy [11]. The most representative cross-sectional methods are BPF (brain parenchymal fraction), FSL-SIENAX (Structural Image Evaluation using Normalization of Atrophy cross-sectional), FreeSurfer, CIVET, and icobrain cross [15]. Methods that can be used in longitudinal analyses are FSL-SIENAX, brain boundary shift integral (BBSI), statistical parametric mapping (SPM), and icobrain long [15]. In clinical trials, SIENA and BPF are the most commonly used methods. The SIENA method (a variant of SIENAX) estimates global and regional brain tissue volumes normalized for the subject's head size [16]. The measurement error reported in this method was 0.2% [17]. BPF is calculated as the ratio of brain parenchyma tissue volume to the total volume contained within the brain surface contour [18]. It is a fully automated method segmentation program with measurement error of less than 0.2% [19]. Icobrain long is a commercial, fully automated method with low measurement error (median error 0.13%) and a strong level of statistical agreement and consistency with SIENA for measuring annualized percentage brain volume change [20]. Most of the widely used longitudinal methods enable only total brain volume change to be measured. A recently published study indicated that icobrain and SPM were valuable methods for longitudinal analysis of whole brain volume and GM atrophy in MS [21]. Development of MRI segmentation and volumetric methods raises the possibility of incorporating longitudinal assessment of WM, GM, and deep GM structures (the thalamus) atrophy in further clinical trials.

The clinical utility of brain atrophy evaluation appears incontestable. Many previous studies have indicated a correlation between brain atrophy, physical disability, and deterioration of cognitive functions [22]. Whole brain and central atrophy are predictors of disability progression, measured with EDSS [23]. Findings from another cross-sectional study indicate that cognitive performance (information processing speed) correlates with deep GM volume, while in a 5-year longitudinal analysis, cortical GM volume was a predictor of cognitive decline [24]. Thalamus volume was observed to be a strong predictor of cognitive impairment (information processing speed and attention) in the early stage of RRMS [25].

Given that brain atrophy is associated with long-term, irreversible disability among patients with MS, it is necessary to determine the influence of DMT on MRI volumetric parameters. The goal of our study was to provide an overview of clinical trials using brain atrophy as an outcome of an immunomodulatory therapy of RRMS. We also assessed whether the neuroprotective effect of DMT is correlated with long-term disability progression.

## 2. Materials and Methods

We performed a literature search in PubMed for studies performed between 2008 and 2019 on RRMS patients that contain brain atrophy/BVL as an outcome of DMT according to PICO search strategies [26]. We used general search terms such as multiple sclerosis, immunomodulatory, disease-modifying drug, brain atrophy, brain volume, BPF, randomized, and RCT. We limited the search with individual drug names (interferon, teriflunomide, fingolimod, natalizumab, cladribine, dimethyl fumarate, ocrelizumab, glatiramer, alemtuzumab, rituximab, siponimod, ozanimod, and placebo). We also searched ClinicalTrials.gov for trials for the following criteria from 2008 to 2019: multiple sclerosis, relapsing-remitting, interventional studies, age 18-64, and phase 3 or 4, including studies with results, active, not recruiting, and completed studies. We included post hoc analysis and interventional, randomized, placebo-controlled or active-controlled trials, and blinded- or assessor-masked trials.

### 2.1. Criteria for considering Studies for Review

Included in our final analysis were retrospective and prospective longitudinal clinical studies that reported atrophy measurement as a primary or secondary outcome of DMT. Another item assessed in our review was clinical disability progression. Electronic searches of the Medline (PubMed) and ClinicalTrials.gov databases for clinical trials published from 01 January 2008 to 01 December 2019 were performed. Two independent raters (MC and JK) reviewed the studies according to the following search design: retrospective and prospective 3-phase placebo-controlled and active-controlled trials with at least 2 years of duration. Only trials with FDA-approved immunomodulatory therapy for RRMS were included. The nonagreement of including or excluding a study was discussed and resolved.

### 2.2. Cohort Size

Studies eligible for inclusion included >100 patients over 18 years old who were diagnosed with RRMS.

### 2.3. MRI Techniques

For inclusion, studies were required to employ at least one of the following MRI measurements: BPF and BVL/change. We included studies with at least two structural MRIs during a minimum of two years of study.

### 2.4. Outcome Measures

The primary or secondary outcome was BPF or BVL/change from baseline to week 96 or from week 24 to week 96 of immunomodulatory treatment.

## 3. Results

**Figure 1:**
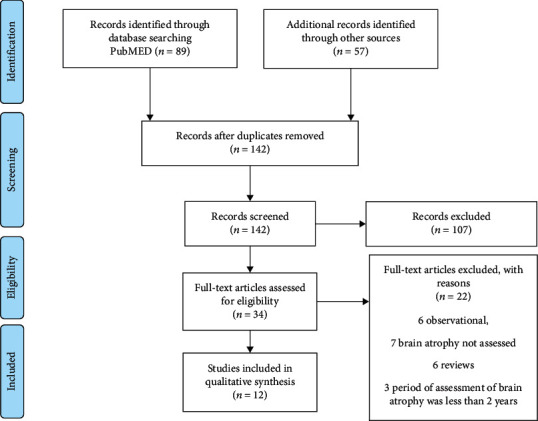
PRISMA 2009 flow diagram [27].

According to the PRISMA statement [27], a total of 146 records were identified, 142 studies were screened for inclusion, and 34 full-text records were assessed for eligibility (Figure 1). Twenty-two studies were excluded due to the following: six studies were observational, six were reviews, brain atrophy was not measured in seven studies, and period of observation was less than 2 years in three studies. Finally, 12 studies were included in the systematic review (Figure 1). All clinical trials were conducted in accordance with the International Conference on Harmonization Guidelines for Good Clinical Practice and Declaration of Helsinki. The protocols were approved by central or local ethics committees, and written consent was obtained.

Quality of data was assessed in the analyzed studies. Evaluation of the risk of bias was performed using RoB 2 [28]; detailed analysis is included in Supplement 1.

### 3.1. Baseline Characteristics

Patient demographics and clinical characteristics were generally similar in the reviewed studies (Supplement 2). The number of patients exceeded 500 in every clinical trial. We observed a similar proportion of females to males in the studied populations. In most clinical trials, there was a comparable mean age; only the FREEDOMS II population included slightly older participants [29]. It should be noted that in clinical trials, CLARITY [30], FREEDOMS I and II [29, 31], and OPERA I and II [32, 33], the population of patients presented with longer mean disease duration than in other studies. Patients in the CARE MS II [34] and CLARITY [30, 35] studies had slightly higher baseline EDSS scores compared to other study populations.

### 3.2. Effect of Studied DMT on Brain Atrophy and Disability

Detailed data illustrating the effect of DMT impact on brain volumetric MRI parameters and the disability of RRMS patients are presented in Table 1.

Alemtuzumab is a humanized monoclonal antibody against CD52 (cluster of differentiation 52), a cell surface marker present on monocytes and lymphocytes. The application of alemtuzumab leads to rapid and long-lasting depletion predominantly of CD-52-bearing B and T cells. Alemtuzumab exhibited a significant reduction in brain parenchymal loss during the 96-week treatment period compared to INF-*β*-1a in both CARE MS I (*p* < 0.0001) [36] and CARE MS II study (*p* = 0.01) [34]. A sustained effect on the reduction of brain atrophy was also observed in an extension of a 5-year follow-up period. Mentioned above, favorable MRI results were associated with a reduction in the risk of sustained accumulation of disability (p =0.0084) [34]. The alemtuzumab group displayed improved MSFC scores compared to the INF-*β*-1a group, but this effect was not significant.

Ocrelizumab is an anti-CD20 antibody that depletes circulating immature and mature B cells. The effector mechanisms of anti-CD20 antibodies are complement-dependent cytotoxicity and antibody-dependent cellular cytotoxicity. Ocrelizumab significantly reduced BVL in over 24-96 weeks of treatment, compared to INF-*β*-1a in the OPERA I study [33], but this effect was not observed in the OPERA II study [32]. In both OPERA I and OPERA II, treatment with ocrelizumab was associated with a significant reduction in the 3-month and 6-month confirmed disability progression. Patients treated with ocrelizumab had a 40% reduction in the risk of clinical disability progression [32, 33]. In the OPERA II, MSFC score improvement in the ocrelizumab group was significant (*p* = 0.004) [32].

Fingolimod, a once-daily oral drug for RRMS, is a sphingosine-1-phosphate (S1P) agonist, binds to 4 of the 5 S1P subtypes and acts as a functional antagonist. Fingolimod interferes with a key S1P mechanism that lymphocytes use to exit lymph nodes [37]. It also enters the central nervous system and affects neurons and supporting glial cells, which express the S1P receptor [37]. Fingolimod in doses of 1.25 mg and 0.5 mg both displayed a significant effect on the reduction of brain volume evaluated in the second year of treatment (*p* < 0.0001 and *p* = 0.0002, respectively) vs. placebo in the FREEDOMS I and FREEDOMS II clinical trials [29, 31]. However, only in FREEDOMS I, a considerable reduction in brain atrophy, was associated with decreased disability progression, measured as 3- and 6-month confirmed disability progression and MSFC score improvement at the second year of treatment [31]. This favorable clinical effect of fingolimod was not observed in the FREEDOMS II study [29].

Teriflunomide is a daily oral immunomodulatory therapy that selectively and reversibly inhibits dihydroorotate dehydrogenase, a key mitochondrial enzyme in the de novo pyrimidine synthesis pathway, leading to a reduction in the proliferation of activated T and B lymphocytes, and limiting their involvement in the inflammatory processes in MS [38]. Teriflunomide at doses of 7 mg and 14 mg exhibited significant effects on annualized brain volume change (*p* = 0.0019 and *p* = 0.0001, respectively), up to a 30% treatment difference compared to the placebo in the TEMSO study [39]. This MRI result was associated with a favorable clinical effect observed among the treated population as a significantly reduced percentage of patients with confirmed disability worsening [40].

Dimethyl fumarate is a twice-daily oral drug that acts by activation of the transcription factor nuclear factor erythroid-derived 2 (Nrf2). Dimethyl fumarate displayed a moderate reduction in the percentage change in brain volume (*p* < 0.05) in the DEFINE study [41]. In the further CONFIRM study [42], the effect on brain atrophy was not observed. The authors did not observe a clinical correlation between the MRI effect on brain volume change and progression of clinical disability.

Ozanimod, a sphingosine 1-phosphate receptor modulator, selectively binds to sphingosine 1-phosphate receptor subtypes 1 and 5 with high affinity. Ozanimod at doses of 0.5 mg and 1 mg significantly reduced whole BVL, cortical GM loss, and thalamic volume loss compared to interferon beta 1-a [43]. Ozanimod did not have as strong an effect on confirmed disability progression as INF-*β*-1a and only had a marginal effect on MSFC score improvement at a dose of 0.5 mg [43].

## 4. Discussion

The aim of our study was to evaluate the effect of DMT on brain atrophy. Our observation is that brain atrophy, measured in clinical trials as brain volume change, is not routinely evaluated as a primary or secondary outcome. In the ClinicalTrials.gov database, we observed that among 318 interventional studies of RRMS with results, only 28 provided brain volume as an outcome, which is about 8.8% [48]. In most of the reviewed clinical trials, authors who employed MRI-derived measures of brain volume used SIENAX, while BPF was assessed in the minority. There was a predominance of whole-brain atrophy assessment; less often, authors used segmentation techniques to analyze regional atrophy (i.e., WM, GM, and thalamic atrophy). There is extensive evidence that cortical atrophy is a more relevant clinical predictor than whole brain atrophy [23]. Cognitive decline in RRMS and primary progressive multiple sclerosis patients is more closely correlated with cortical and deep GM atrophy [24]. These findings should encourage clinicians to use GM atrophy as a biomarker of neurodegeneration to perform more personalized decisions while choosing treatment options. It is worth noting that major limitation of studies on atrophy nowadays is the heterogeneity of evaluated parameters (BVL, BPF, cortical atrophy, WM atrophy, and regional atrophy) without a clear predominance of one on another.

We found limited information about the MRI scanner, field strength, and changes in the scanner during clinical trials. There is literature indicating that brain parenchymal volumes measured from scans obtained at 1.5 T may be biased, due to low tissue contrast compared to scans obtained at 3 T scanners; 1.5 T scanners have a tendency to overestimate the brain parenchymal volume [49].

We observed that there was a different timeframe of brain atrophy evaluation. In some studies, atrophy measurement was performed from pretreatment baseline to 48 weeks or 96/144 weeks of therapy; in other studies (e.g., CLARITY and DEFINE), authors used the 24th week of treatment as a reference. The aim of such evaluation was to exclude the “pseudo-atrophy” phenomenon. A trial period of 1.5-2 years seems to be relatively short to observe the effect on brain atrophy. A longer follow-up time would likely allow brain volume changes to be a more valuable marker of neurodegeneration. In most of the analyzed clinical trials, patients in the placebo and active comparator arm were switched to the treatment arm after 1 or 2 years of treatment. Such an arrangement of randomized clinical trials is associated with some bias, which should be taken into consideration during drug efficacy analysis. For this reason, analysis of DMT's efficacy, when extracted from multiple comparative trials with longer follow-up periods, may be more valid. Other crucial factors influencing DMT's therapeutic results are disease duration and previous treatment of MS.

We also observed heterogeneity in the measurement of the clinical effect of DMTs. Therapeutic response to DMTs was assessed as the percentage of patients with confirmed disability worsening/progression in 3 or 6 months, but also as confirmed disability improvement or percentage of patients with NEDA at different time points. Such a variety of clinical outcomes makes comparisons of DMT's efficacy difficult.

More severe structural damage at baseline predicts clinical decline during follow-up [24]. In some clinical trials (e.g., OPERA, FREEDOM, and CLARITY), we observed that the patient population had a longer disease duration and was older, which could be associated with more pronounced baseline brain atrophy. In the reviewed clinical trials, the DMT group revealed that reduction of brain atrophy is consistent with lowering the risk of clinical disability progression. This observation was positive for ocrelizumab, alemtuzumab, pegylated interferon *β* 1-a, teriflunomide, and cladribine. Fingolimod showed significant effects on brain atrophy in both the FREEDOMS I and FREEDOMS II study. The effect on disability progression was observed in FREEDOMS I but not in FREEDOMS II. Participants in FREEDOMS II were older and had a longer duration of disease as compared to that in other studies. This observation suggests that some DMTs could be more clinically effective when applied early in the course of RRMS. Dimethyl fumarate which acts on brain atrophy to a very small extent, also demonstrated no significant effect on disability progression. In the RADIANCE study, ozanimod demonstrated significant effects on brain atrophy, GM atrophy, and thalamic atrophy but was not associated with a positive influence on disability progression and the MSFC score. Our findings from the analyzed trials suggest that BVL is only partially responsible for disability worsening; other factors are annualized relapse rate and baseline brain volume. Currently, there is no validated tool to assess whole brain, WM, and GM atrophy at the individual level. It also remains to be determined what is an appropriate pathological cut-off is considering annualized whole brain atrophy. NEDA-4 criteria indicate 0.4, but recent work by Opfer et al. suggests that BVL ≥ 0.94% takes into account technique measurement error and short-term biological brain volume fluctuations [20, 50]. This review suggests that evaluation of brain atrophy should be used as an outcome in clinical trials and assessed routinely to track treatment responses. It should also be considered in combination with other clinical data (i.e., patient age, duration of disease, baseline EDSS, and steroid use). This observation should encourage the development and validation of MRI-derived volumetric techniques destined for automatic measurement of MS brain atrophy comprising routine measurement of whole brain atrophy, WM, and GM matter atrophy, including cortical and deep GM. Such a widespread method, used in daily practice, would help identify patients at risk of clinical decline, allowing accurate decision-making in MS therapy. There is also a need to standardize the time periods and methods of evaluating the effectiveness of therapeutic interventions, with routine assessment of cognitive function. In the majority of reviewed clinical trials, the annualized relapse rate was evaluated as the primary outcome. Disease progression measured as EDSS change is internationally accepted and is the most widely used endpoint in clinical trials. EDSS changes by 1.0 points from baseline, EDSS less than or equal to 5.5, and EDSS 0.5 points over baseline 5.5 are commonly recognized as clinically relevant increases in disability, when persistent for at least 3 or 6 consecutive months [51]. In most of the reviewed clinical trials, confirmed disability progression was used as a secondary outcome.

A novel clinical marker for DMTs' effectiveness, more closely associated with neurodegeneration, is percent of patients with progression independent of relapse activity. Another instrument for disability measurement, usually performed as a secondary outcome, was the MSFC score change, which could provide more information regarding upper extremity function and cognitive skills. Known limitations of MSFC include the interpretation of Z-scores, the learning effect of the 9-Hole Peg Test and Paced Auditory Serial Addition Test (PASAT), and low patient acceptance of PASAT [51].

To conclude, it is reasonable to incorporate BVL as an outcome in clinical trials to evaluate intervention effectiveness at the MS population level. Recognizing the fact that the comparison of different RCT in the extrapolation of a single endpoint is always a source of bias. Despite some methodological aspects, it should also be considered at the individual level in treatment decision-making.

Despite our systematic findings, this review is not free of limitations. First, we did not take into account duration of disease and previous treatments prior to the inclusion in the clinical trial. Second, our analysis only covered the last decade of DMT clinical trials for RRMS.

## Figures and Tables

**Table 1:** Brain atrophy outcome data.

Intervention clinical trial	BVL timeframe	Percentage change of brain volume	Disability effect timeframe	Effect on disability
*CARE MS I* [36] Group 1, alemtuzumab Group 2, IFN-*β*-1a s.c	At wk 48 (y1) At wk 96 (y2)	Significant reduction in median change in BPF -0.867 in the alemtuzumab group vs. -1.488 in the IFN*-β*-1a group (*p* < 0.0001).	wk 96 (y2)	30% reduction of risk of 6-month CDA, the effect on sustained 6-month disability accumulation and change in EDSS was not significant. Significant improvement in MSFC score, mean change 0.15 in alemtuzumab group vs. 0.07 in IFN-*β*-1a (*p* = 0.01).

*CARE MS II* [34] Group 1, alemtuzumab Group 2, IFN-*β*-1a s.c	At wk 96 (y2)	Significant reduction in median change in BPF -0.615 in the alemtuzumab group vs. -0.810 in the interferon IFN-*β*-1a s.c group (*p* = 0.01).	wk 96 (y2)	42% risk reduction of 6-month CDA (*p* = 0.0084) in alemtuzumab compared to IFN-*β*-1a group. 28.2% alemtuzumab-treated patients vs. 12.93% IFN-*β*-treated patients (95% CI) with sustained reduction of disability for 6-month. Mean MSFC score improved from baseline by 0.08 in alemtuzumab-treated patients vs. worsened from baseline by -0.04% in IFN-*β*-1a-treated patients (*p* = 0.02).

*OPERA I* [32, 33] Group 1, ocrelizumab Group 2, INF-*β*-1a	wk 24-96 (y2)	Significant difference in mean percentage BVC -0.57 in the ocrelizumab group (95% CI) vs. −0.74 in the INF-*β*-1a group (95% CI), difference in rate 22.8% (*p* = 0.004).	wk 24-96 (y2)	3-month CDP 9.8% in ocrelizumab-treated group vs. 15.2% in INF-*β*-1a group (95% CI, 0.45–0.81) (*p* < 0.001). 6-month CDP 7.6% in ocrelizumab-treated group vs. 12% % in INF-*β*-1a group (95% CI, 0.43–0.84) (*p* = 0.003). In OPERA I, MSFC score between ocrelizumab group and INF 1a group was not significant In OPERA II MSFC score MSFC 0.28 in ocrelizumab-treated group vs. 0.17 in INF-*β*-1a-treated group. Difference (95% CI) 0.11 (0.03 to 0.18) (*p* = 0.004).
*OPERA II* [32, 33] Group 1, ocrelizumab Group 2, INF-*β*-1a	wk 24-96 (y2)	Mean percentage change in brain volume was -0.646 in ocrelizumab group (95% CI) vs. 0.75 in INF-*β*-1a group. Difference in rate 14.9% (*p* = 0.09) was not significant.		

*ADVANCE* [44] Group 1, Peg-IFN-*β*-1a Q4w Group 2, Peg-IFN-*β*-1a Q2w Group 3, delayed treatment	wk 24-96 (y2)	Significant reduction in mean percent change in whole brain volume 0.88% (*p* < 0.05) in IFN-*β*-1a Q4w group vs. -0.8% in the IFN-*β*-1a Q2w group (p <0.001) vs. delayed treatment group -1.01	BL-wk 96	Significant difference in percentage of patients with clinical NEDA in IFN 1-a Q4w-treated group 64.2% (*p* = 0.016) and IFN 1-a Q2w-treated group 71.3% (*p* = 0.0001) vs. delayed treatment.

*FREEDOMS I* [31, 45] Group 1, fingolimod 1,25 mg Group 2, fingolimod 0.5 mg Group 3, placebo	BL–wk 96 (y2)	Significant reduction in men BVC in 1.25 mg fingolimod group -0.89 (*p* < 0.001) vs. in fingolimod 0.5 mg -0.84 (*p* < 0.001) vs. placebo group -1.31	BL-wk 96	About 40% reduction of risk of 6-month CDP in both 1.25 mg and 0.5 mg fingolimod group. Fingolimod demonstrated significant effect on disability: absence of 3-month CDP was 83.4% ± 1.9 in fingolimod 1.25 mg group (*p* = 0.01) and 82.3% ± 1.9 in 0.5 mg fingolimod group (*p* = 0.03) and 75.9% ± 2.2 in placebo group. Absence of 6-month CDP was 88.5% (*p* = 0.004) in fingolimod 1.25 mg group (*p* = 0.01), and 87.5% in 0.5 mg fingolimod group (*p* = 0.01) vs. 81.0% in placebo group. Significant improvement of MSFC Z-score was observed in 1.25 mg fingolimod group 0.01 ± 0.40 (*p* = 0.02) and in 0.5 mg fingolimod group 0.03 ± 0.39 (*p* = 0.01) vs. placebo group with MSFC worsening −0.06 ± 0.57.

*FREEDOMS II* [29] Group 1, fingolimod 1,25 mg Group 2, fingolimod 0.5 mg Group 3, placebo	BL–wk 96 (y2)	Significant reduction in BVC in fingolimod 1.25 mg -0.595 vs. -0.858 in fingolimod 0.5 mg vs. -1.279 in placebo group. Treatment difference vs. placebo in fingolimod 1.25 mg was -0.63% (*p* < 0.0001), in fingolimod 0.5 mg was -0.41% (*p* = 0.0002)	BL-wk 96	Fingolimod 1.25 mg and 0.5 mg had no significant effect on 3- and 6-month confirmed disability progression and MSFC Z-score at 96 week (y2) comparing to placebo.

*TEMSO* [39] Group 1, teriflunomide 7 mg Group 2, teriflunomide 14 mg Group 3, placebo	BL-wk 96 (y2)	Annualized median percentage BVC was -0.94 in teriflunomide 7 mg group, 0.9 in teriflunomide 14 mg group, and -1.29 in placebo group. Treatment difference vs. placebo 27.6% in teriflunomide 7 mg group (*p* = 0.0019) and 30.6% in teriflunomide 14 mg group (*p* = 0.0001)	BL-wk 96	Teriflunomide 14 mg group displayed significant reduction in 3-month confirmed disability worsening (*p* = 0.004) and 6-month confirmed clinical worsening (*p* = 0.006) measured from baseline to wk 96.

*CLARITY* [30] Group 1, cladribine 3.5 mg/kg Group 2, cladribine 5.25 mg/kg Group 3, placebo	wk 24-96 (y2)	Mean percentage of BVC −0.77 (*p* = 0.02) in cladribine 3.5 mg/kg group and −0.77 (*p* = 0.02) in cladribine 5.25 mg/kg group vs. −0.95 in placebo group	BL-wk 96	43% risk reduction of 3-month sustained EDSS change in 3.5 mg cladribine group vs. placebo CI: (0.518-0.934) (*p* = 0.015) and 36% risk reduction of 3-month sustained EDSS change in 5.25 mg cladribine group vs. placebo CI: (0.553-0.977) (*p* = 0.033).

*DEFINE* [41] Group 1, DMF BID Group 2, DMF TID Group 3, placebo	BL-wk 96 (y2)	Only dimethyl fumarate twice daily (DMF BID) showed significant reduction in median percentage change in whole brain volume at week 96, -0.64% in DMF BID group vs. -0.81 in placebo group, treatment difference vs. placebo 21% (*p* < 0.05)	Ø	Not evaluated

*CONFIRM* [46, 47] Group 1, DMF BID Group 2, DMF TID Group 3, glatiramer acetate Group 4, placebo	BL-wk 96 (y2)	No significant effect of treatment with dimethyl fumarate on median percentage change in whole brain volume	At wk 96	No significant correlation between brain parenchymal volume change and change in EDSS

*RADIANCE* [43] Group 1, ozanimod 0.5 mg Group 2, ozanimod 1 mg Group 3, IFN beta-1a	BL-wk 96 (y2)	Significant reduction in percent of whole brain volume loss in 0.5 mg ozanimod group (*p* = 0.0002) and in 1 mg ozanimod group (*p* < 0.0001) vs. IFN 1-a. Significant reduction in cortical gray matter volume loss in 0.5 mg ozanimod group (*p* < 0.0001) and in 1 mg ozanimod group (*p* < 0.0001) vs. IFN beta-1a. Significant reduction in thalamic volume loss in 0.5 mg ozanimod group (*p* < 0.0133) and in 1 mg ozanimod group (*p* < 0.0004) vs. IFN beta-1a	At wk 96	No significant difference between group treated with 0.5 mg and 1 mg ozanimod and in IFN-*β*-1a in reduction of 3- and 6-month CDP or change of MSFC Z-scores.

Abbreviations: wk: week; y: year; Q4w: every 4 weeks; Q2w: very 2 weeks; BPF: brain parenchymal fraction; BVC: brain volume change; DMF BID: delayed-release dimethyl fumarate twice daily; DMF TID: delayed-release dimethyl fumarate three times daily; INF-*β*-1a: interferon beta-1a; BVL: brain volume loss; s.c: subcutaneous; DMT: disease-modifying therapies; EDSS: Expanded Disability Status Scale; BL: baseline; CDA: Confirmed Disability Accumulation; CDP: confirmed disability progression; MSFC: Multiple Sclerosis Functional Composite; Clinical-NEDA: no relapses and no onset of 12-week CDP—increase in the EDSS score of *EDSS* ≥ 1.0 in patients with baseline score of ≥1.0, or an increase of 1.5 points in patients with a baseline score of 0, confirmed after 12 or 24 weeks.

## Data Availability

The data supporting this review are from previously reported studies and datasets, which have been cited. The processed data are available in the supplementary files/from the corresponding author upon request. Author (magdalena.chylinska@gumed.edu.pl) will make data available on reasonable request.

## Supplementary Materials

Supplementary 1 Supplement 1 Risk of bias according to RoB2.

Supplementary 2 Supplement 2 Patients' demographic and clinical characteristics.
