# Supplementary material for: Brain Atrophy as an Outcome of Disease-Modifying Therapy for Remitting-Relapsing Multiple Sclerosis
Source: Mult Scler Int. 2023 Aug 31;2023:4130557. doi: 10.1155/2023/4130557 (PMC10484652; doi:10.1155/2023/4130557)
Supplement: Supplementary 1 — Supplement 1 Risk of bias according to RoB2. [file 4130557.f1.docx]

**Table 1. Risk of bias**

| **Study** | **Domain** | | | | | |
| --- | --- | --- | --- | --- | --- | --- |
|  | **Randomization process** | **Deviations from intended interventions** | **Missing outcome data** | **Measurement of the outcome** | **Selection of the reported result** | **Overall Bias** |
| **CARE-MS I NCT00530348** |  |  |  |  |  |  |
| **CARE-MS II NCT00548405** |  |  |  |  |  |  |
| **OPERA I**  **NCT01247324** |  |  |  |  |  |  |
| **OPERA II NCT01412333** |  |  |  |  |  |  |
| **ADVANCE NCT00906399** |  |  |  |  |  |  |
| **FREEDOMS I NCT00289978** |  |  |  |  |  |  |
| **FREEDOMS II NCT00355134** |  |  |  |  |  |  |
| **TEMSO**  **NCT00134563** |  |  |  |  |  |  |
| **CLARITY**  **NCT00213135** |  |  |  |  |  |  |
| **DEFINE**  **NCT00420212** |  |  |  |  |  |  |
| **CONFIRM NCT00451451** |  |  |  |  |  |  |
| **RADIANCE**  **NCT02047734** |  |  |  |  |  |  |

Risk of bias: low   , some concerns , high risk of bias
