# Supplementary material for: Brain Atrophy as an Outcome of Disease-Modifying Therapy for Remitting-Relapsing Multiple Sclerosis
Source: Mult Scler Int. 2023 Aug 31;2023:4130557. doi: 10.1155/2023/4130557 (PMC10484652; doi:10.1155/2023/4130557)
Supplement: Supplementary 2 — Supplement 2 Patients' demographic and clinical characteristics. [file 4130557.f2.docx]

| Study  Clinical trial number | Author | Intervention | Randomized  Patients, allocation | Age mean in subgroups  (SD), y | | Female,  n (%) | Time since diagnosis,  mean (SD),y | | Baseline EDSS, mean (SD) | |
| --- | --- | --- | --- | --- | --- | --- | --- | --- | --- | --- |
| CARE-MS I  NCT00530348 | Cohen et al 2012 (26) | Group 1, Alemtuzumab 12mg i.v | 581, 2:1 | Group 1 | 33.0 (8.03) | 243 (64.6) | 2.0 (1.32) | | 2.0 (0.79) | |
|  |  | Group 2, INFβ-1a s.c |  | Group 2 | 33.2 (8.48) | 122 (65.2) | 2.1 (1.36) | | 2.0 (0.81) | |
| CARE-MS II  NCT00548405 | Coles et al 2012 (27) | Group 1, Alemtuzumab 12mg i.v | 840, 2:2:1 | Group 1 | 34.8 (8.36) | 120 (70.6) | 3.8 ( 0.2-14.4) | | 2.7 (1.17) | |
|  |  | Group 2, Alemtuzumab 24mg i.v |  | Group 2 | 35.1 (8.40) | 120 (70.6) | 3.7 (0.2-16.9) | | 2.7 (1.26) | |
|  |  | Group 3, INFβ-1a s.c |  | Group 3 | 35.8 (8.77) | 131 (64.9) | 4.1 (0.4-10.1) | | 2.7 (1.21) | |
| OPERA I  NCT01247324 | Kappos et al 2017 (28) | Group 1, Ocelizumab | 821, 1:1 | Group 1 | 37.1 (9.3) | 270 (65.9) | 6.74 (6.37) | | 2.86 (1.24) | |
|  |  | Group 2, INFβ-1a |  | Group 2 | 36.9 (9.3) | 272 (66.2) | 6.25 (5.98) | | 2.75 (1.29) | |
| OPERA II  NCT01412333 | Kappos et al 2017 (28) | Group 1, Ocelizumab | 835, 1:1 | Group 1 | 37.2 (9.1) | 271 (65.0) | 6.72 (6.10) | | 2.78 (1.30) | |
|  |  | Group 2, INFβ-1a |  | Group 2 | 37.4 (9.0) | 280 (67.0) | 6.68 (6.13) | | 2.84 (1.38) | |
| ADVANCE  NCT00906399 | Calabresi et al. 2014 (30) | Group 1, Peginterferon 125µg s.c beta 1a Q4w | 1516, 1:1:1 | Group 1 | 36.4 (9.87) | 352 (70.4) | 3.4 (4.4) | | 2.48 (1.24) | |
|  |  | Group 2, Peginterferon 125µg s.c beta 1a Q2w |  | Group 2 | 36.9 (9.79) | 361 (70.5) | 4.0 (5.1) | | 2.47 (1.25) | |
|  |  | Group 3, Placebo |  | Group 3 | 36.3 (9.74) | 358 (71.6) | 3.5 (4.6) | | 2.44 (1.18) | |
| FREEDOMS I  NCT00289978 | Kappos et al. 2010 (31) | Group 1, Fingolimod 0.5mg | 1272, 1:1:1 | Group 1 | 36.6 (8.8) | 296 (69.9) | 8.0 (6.6) | | 2.3 (1.3) | |
|  |  | Group 2, Fingolimod 1.25mg |  | Group 2 | 37,4 (8.9) | 295 (68.8) | 8.4 (6.9) | | 2.4 (1.4) | |
|  |  | Group 3, Placebo |  | Group 3 | 37.2 (8.6) | 298 (71,3) | 8.1 (6.4) | | 2.5 (1.3) | |
| FREEDOMS II  NCT00355134 | Kappos et al.  2014 (32) | Group 1, Fingolimod 0.5mg | 1083, 1:1:1 | Group 1 | 40.6 (8.4) | 275 (77) | 10.4 (8.0) | | 2.4 (1.3) | |
|  |  | Group 2, Fingolimod 1.25mg |  | Group 2 | 40.9 (8.9) | 281 (76) | 10.8 (8.2) | | 2.5 (1.3) | |
|  |  | Group 3, Placebo |  | Group 3 | 40.1 (8.4) | 288 (81) | 10.6 (7.9) | | 2.4 (1.3) | |
| TEMSO  NCT00134563 | O’Connor et al.  2011 (33) | Group 1, Teriflunomide 7mg | 969, 1:1:1 | Group 1 | 37.5 (9.0) | 254 (69.6) | 5.29 (5.36) | | 2.61 (1.29) | |
|  |  | Group 2, Teriflunomide 14mg |  | Group 2 | 37.8 (8.2) | 254 (70.9) | 5.59 (5.44) | |  |  |
|  |  | Group 3, Placebo |  | Group 3 | 38.4 (9.0) | 275 (75.8) | 5.13 (5.59) | |  |  |
| CLARITY  NCT00213135 | Giovannoni et al. 2010 (34) | Group 1, Cladribine 3.5mg/kg | 1326, 1:1:1 | Group 1 | 37.9 (10.3) | 298 (68.8) | 7.9 (0.3-42.3)^a^ | | 2.8 (1.2) | |
|  |  | Group 2, Cladribine 5.25mg/kg |  | Group 2 | 39.1 (9.9) | 312 (68.4) | 9.3 (04-35.2)^a^ | | 3.0 (1.4) | |
|  |  | Group 3, Placebo |  | Group 3 | 38.7 (9.9) | 288 (65.9) | 8.9 (0.4-39.5)^a^ | | 2.9 (1.3) | |
| DEFINE  NCT00420212 | Arnold et al. 2012 (35) | Group 1, DMF BID^b^ | 1237, 1:1:1 | Group 1 | 38.3 (9.31) | 136 (77) | 5.6 (5.54) | | 2.3 (1.17) | |
|  |  | Group 2, DMF TID^c^ |  | Group 2 | 38.5 (8.59) | 140 (76) | 5.0 (4.96) | | 2.3 (1.19) | |
|  |  | Group 3, Placebo |  | Group 3 | 38.3 (9.16) | 141 (78) | 6.0 (5.76) | | 2.5 (1.25) | |
| CONFIRM  NCT00451451 | Miller et al 2015 (36) | Group 1, DMF BID^b^ | 1417,1:1:1:1 | Group 1 | 38.5 (8.9) | 118 (70) | 4.9 (5.1) | | 2.5 (1.1) | |
|  |  | Group 2, DMF TID ^c^ |  | Group 2 | 38.2 (9.7) | 121 (71) | 4.6 (5.2) | | 2.5 (1.2) | |
|  |  | Group 3, GA^d^ |  | Group 3 | 36.8 (8.8) | 123 (70) | 4.4 (4.7) | | 2.5 (1.3) | |
|  |  | Group 4, Placebo |  | Group 4 | 36.6 (9.1) | 116 (69) | 4.8 (5.0) | | 2.5 (1.1) | |
| RADIANCE  NCT02047734 | Cohen et al 2019 (39) | Group 1 Ozanimod 0.5 mg po QD | 1320; 1:1:11 | Group 1 | 35.4 (8.8) | 287 (65.4) | | 3.5 (4.21) | | 2.5 (1.17) |
|  |  | Group 2, Ozanimod 1mg po QD |  | Group 2 | 36.0 (8.9) | 291 (67.2) | | 4.0 (5.17) | | 2.6 (1.15) |
|  |  | Group 3, Interferon beta-1a IM once a week |  | Group 3 | 35.1 (9.1) | 304 (68.9) | 3.6 (4.61) | | 2.5 (1.15) | |

**Table 2 Patients demographic and clinical characteristics**

**Q4w**: every 4 weeks, **Q2w**:every 2 weeks, ^a^ range, **DMF BID**: delayed-release dimethyl fumarate twice daily, **DMF TID**: delayed-release dimethyl fumarate three times daily, **GA**: glatiramer acetate, ^b^Median (P25, P75), **QD**: once a day, **QWK**: once a week, **INFβ-1a**: interferon beta-1a
